# Determinants of client satisfaction with family planning services in public health facilities of Jigjiga town, Eastern Ethiopia

**DOI:** 10.1186/s12913-019-4475-5

**Published:** 2019-09-02

**Authors:** Aregawi Gebreyesus

**Affiliations:** School of Public Health, Department of Epidemiology, Mekelle University, P.O.Box 1871, Mekelle, Ethiopia

**Keywords:** Client satisfaction, Family planning, Jigjiga

## Abstract

**Background:**

Client satisfaction is the best indicator of quality service provision and has been found to correlate with continuity of care perceived by the client. The measurement of client satisfaction helps in understanding willingness and decisions to return for future services. Thus, this study aimed at assessing the level and factors associated with client satisfaction of FP services among family planning users.

**Methods:**

An institutional based cross-sectional study was conducted on 492 family planning users in Public health facilities of Jigjiga town, Eastern Ethiopia from February 10 to March 10, 2017. Participants were chosen by systematic random sampling and interviewed immediately after having received family planning service using via a structured and pre-tested questionnaire. The data were entered into Epi Info 7 and then exported to SPSS 20 for analysis. All associations with client satisfaction were tested for statistical significance with alpha set at the 0.05 level.

**Result:**

The findings of this study showed that the overall client satisfaction with FP services among FP users of public health facilities of Jigjiga town was 41.7% with 95% CI of (37 − 46.1%). Knowledge on FP [adjusted odds ratio, AOR (95% CI) = 0.037 (0.019–0.072)], demonstrate how to use the method [AOR (95% CI) = 16.1 (8.4–30.7)], describing side effect of the method [AOR (95% CI) = 2.4 (1.41–4.23)] and distance of FP user’s home to health facility [AOR (95% CI) = 0.24 (0.14–0.42)] were found to be significantly associated with the client satisfaction of FP services.

**Conclusions:**

The overall client satisfaction with FP services was relatively low. Knowledge of FP, demonstrating how to use the method, describing the side effect of the method and distance of FP user’s home to health facilities were found to be factors that influence client satisfaction with family planning services. Thus, efforts should be made to improve on client interaction especially on the information given and knowledge of clients.

## Background

Family planning is a conscious decision by individuals or couples to choose for themselves when to start having children, how many children to have, how to space them or when to stop having children by using (modern) contraception and natural methods [1, 2]. Utilization of modern family planning services in 3rd world countries have improved significantly from the time when the first programs were begun in the 1950s when contraceptive products were limited to barrier methods. Since then, FP (family planning) has been recognized as a basic component of reproductive health. Better public acceptance of family planning has led to an improved a range of mechanisms for providing FP services [1].

In Africa (primarily in Sub-Saharan Africa), the contraceptive utilization remains low and fecundity, fertility rate, and unmet need for modern contraceptives are high [2, 3]. However, at this moment, the progress of using modern contraceptive method is estimated to be fast in under developed countries mainly in Africa. Between 2017 and 2030, utilization of modern contraceptive is expected to increase from 43 to 56% in Eastern Africa. Globally, above 10% married women have an unmet need for modern contraceptive; that is to say, Percentage of women who are not perinatal amenorrhea and are considered fecund and want to delay their following birth for two or more years or stop childbirth overall but are not by means of modern contraceptive method [2, 4]. In Africa, 20% married women have an unmet need for modern contraceptive. Conversely, based on the UNs (United States) estimation, the total of women with an unmet need for modern contraceptive is predicted to drop to 139 million in 2030 globally from 142 million that was in 2017. The largest declines are expected in Eastern Africa, estimated to decline from 22% in 2017 to 16% in 2030 [5].

Even though modern contraceptive use in married Ethiopian women has gradually increased over the last 15 years, from 6, 13.9, 21, and 40% in 2000, 2005, 2011, and 2016 respectively. However when we come to the Somali region eastern part of Ethiopia didn’t increase yet and it was 2.4, 2.7, 3.8 and 1.4% respectively. There is no a study carried out in Somali region particularly, but the study carried out in Afar (in other pastoralist society), “religious and needs of more children” showed as predictors. When we compare with this, the utilization of modern FP methods in sexually active unmarried women was higher than married women. Based on EDHS of 2016, 55 and 6% of sexually active unmarried women used modern FP methods in Ethiopia and Somali region respectively [5–8]. Hence, the unmet need of FP in this region is 24% [9] and based on the study carried out in Jigjiga town in 2017, the unmet need of FP in that town was 22.6% [10].

It is obvious that, FP plays a critical role in the health of women and the family. It also has a great role on family’s economic and social situations. It improves health (biological, social, mental and psychological), decreases poverty, it empowers women and it has a great contribution in the community’s socio-economic circumstances [1, 9]. Then and there today, interest in FP is increasing gradually. Purpose of FP services should be to deliver safe and good quality service provision to those individuals most in need, in a way that is suitable to FP users served while also efficient in their use of resources [1]. Considering that, FP2020 identifies and recommends all FP users’ right to the highest quality of service; furthermore, existing evidence point out that poor-quality service provisions are the main obstacles to initiation and continual use of modern FP worldwide. To fulfill clients’ need and to intervene acceptable interventions, it needs further additional studies. Ensuring client satisfaction is a basic thing to increase new FP users and in reducing discontinuation [3].

Client satisfaction has been found to correlate with continuity of care perceived by the client [10]. It is considered an indicator of quality service provision [11–13]. Client satisfaction is a relative phenomenon, which embodies the clients perceived need, his/her expectations from the health system, and experience of health care [16, 17]. Client satisfaction has been described as a key to clients’ decisions to use and to continue using services or willingness to return for future services. Satisfaction evaluation is an excellent opportunity to involve clients in the process of assessing programs from users’ perspective and is recognized as a component of quality of care [14, 15]. Measurement of Client satisfaction helps in understanding patients‘experiences of health care, identifying problems and evaluation of quality service provision and health care [16]. Satisfaction is a best response to the discrepancy between previous expectations and perceived performance after consuming services. It is a general attitude formed based on the client’s experience after getting health service and reflects how much the client likes/dislikes a service after experiencing it. The overall satisfaction with health care services is one of the best predictors of client willingness to continue the services [21].

Several studies pointed out that client satisfaction is influenced by different factors. Those are; socio-demographic factors: education (the educated women were more satisfied), occupation (housewives were more satisfied than employers) and age (women with the age of 20–29 were more satisfied) of the client [13, 17], health facilities factors (cleanness of the clinic, convenience of opening hours, and waiting time) [13, 14, 17–19], interpersonal factors (having bad obstetric history, knowledge and attitude, and side effect experiencing) [19, 20], and client-provider factors (privacy and information given) [17, 21–23]. However, there is a scarce of information on client satisfaction with FP services in eastern Ethiopia specifically in the Somali region. Based on EDHS 2011 and 2016, the types of FP methods commonly offered to clients in Jigjiga public health facilities were only the temporary methods: oral contraceptive pills, Injectable, implants and IUCD. Therefore, this study aimed to assess client satisfaction with FP service provision and its determinants among FP user women in public health facilities of Jigjiga town, eastern Ethiopia. Hence, this study was aimed to assess the level and factors associated with client satisfaction of FP services in public health facilities of Jigjiga town, eastern Ethiopia.

## Methods

### Study setting and design

The study was conducted in Jigjiga town, Eastern Ethiopia from February 10 to March 10, 2017. Jigjiga is the capital city of the Somali region and is located in Eastern 650 km from Addis Ababa, the capital city of Ethiopia. According to the Central Statistical Agency of Ethiopia (CSA) 2007 census report, the town has a population of 277,560, of which 128,268 (46.2%) are females. The town has one general hospital and 3 health centers. According to the town health office report, the utilization of modern family planning in Jigjiga town is nine (9%) and all public health facilities of the town provide family planning services. An institutional based cross-sectional study was employed to assess status o and factors associated with client satisfaction of family planning services among FP users in public health facilities of Jigjiga town, eastern Ethiopia.

### Study population

Women who were in the reproductive age (15–49 years) and who received FP services in public health facilities of Jigjiga town were study and target population of this study. All women who were family Planning users at the time of the data collection period (from February 10 to March 10, 2017) were included in the study. However, Staff of the health facilities who were FP service users (to minimize bias related to conflict of interest) was excluded from the study.

### Sample size and procedure

The sample size calculation was calculated using associated factors of client satisfaction with FP services in different studies done on previous time. Sampling was determined based on the double proportion formula on the software of Epi Info StatCalc version 7 after considering the following assumptions; 95% confidence interval (CI), 80% power, 1:1 ratio of exposed to non-exposed group, odds ratio (OR) of 1.9 and taking the proportion of 60% Perceived sufficiency of consultation as a factor for client satisfaction with FP services, and an expected non-response rate of 10% [17]. Finally, 495 study participants were selected using systematic random sampling. The proportional allocation was made for 4 public health facilities of the town (Karamara hospital: 373; Jigjiga health center: 56; Aayar daga health center: 37; and Hanti shacab health center: 28) and every second FP users were taken for the interview.

### Data collection tool and technique

The source of this instrument tool was adopted from previous studies [13, 14, 18–21]. The Data collection was done using a validated and pretested structured questionnaire that translated into Somali and Amharic (the local languages). After the data collectors introduced with clients who used FP service, explained the purpose and benefits of the study and then informed written consent was obtained from each participant. And it was collected through face-face interviews of FP users at the exit of the FP service clinic by six (6) data collectors who were diploma midwives and two supervisors after 3 days training. To minimize observational bias the participants were informed well about the risks, benefits, confidentiality and the right of stopping/rejecting their response any time during the interview.

### Variables and measurements

The outcome variable for this study was **client satisfaction with FP services**. The outcome variable measured using six questions measured clients’ satisfaction levels, six used a Likert scale (three categories): agree (score 1), neutral (0) and disagree (0). The questions were related to **willingness to come back again to the facility**, **willingness to recommend the facility to someone else**, **whether the client was provided with all the required information**, whether s/he **felt free to ask questions** and was **treated with respect**. An additional question: whether the **respondent’s health needs had been met** was also included. The likert scale questions were taken directly from the study conducted in Kenya and in that study, the internal consistency and reliability used for computing the satisfaction score were assessed by calculating Cronbach’s α, which was found to be high (> 0.7), which was 0.78. And then the questionnaire also pre-tested before the actual data collection [18]. Finally, by saying above/equal and below the mean the response was categorized in to “Satisfied (coded as 1)” and “Unsatisfied coded as 0)” respectively.

The independent variables include socio-demographic factors (age, educational status, marital status, religion, ethnicity, occupation, average income status), health facility related characteristics (frequency of visit, type of public health facility, received their preferred method, method that the client was using, distance from home, time of consultation, opening hours convenience, waiting time, and feeling on waiting time), information given and client-provider interaction factors (privacy, information is given, informed about other methods, STI checked, pregnancy checked, check medical, surgical and other problem, asking questions, and communication of the provider), and other interpersonal characteristics.

#### Good knowledgeable of FP

If the participants scored above the mean of 8 knowledge questions. The knowledge of the study participants was measured using eight questions: the women were asked use of FP (pregnancy prevention, STI prevention, for no of family planning), types of FP methods, source of information (health professional, husband/ relatives, friends/neighbors, media), to describing side effects, to tell time of starting of the method they were using, asking who should be decider of FP method (husband, wife, husband and wife), to tell duration of the method they were using, and to describing apart from the regular return conditions.

### Data processing and analysis

The data were first coded, entered using the double data entry by two data clerks and consistency of the entered data was cross checked by comparing the two separately and cleaned using EpiData version 3.1. And then, it exported to the SPSS statistical software version 20.0 for analysis. Descriptive statistical analysis such as simple frequencies, measures of central tendency and measures of variability were used to describe the characteristics. Then the information was presented using frequencies, summary measures, tables, and figures. For assessing client satisfaction with FP services, six Likert scale questions were used. Each question has the alternative “Agree” (score as 1), “Neutral” (score as 0) and “Disagree” (score as 0). Later, the responses were dichotomized into “Satisfied,” if the client reported “Agree” with the FP services received or provided and “Unsatisfied,” if the client reported either “Neutral” or “Disagree” with FP services received or provided.

In the bivariate regression analysis, those variables with a *p*-value ≤0.25 were entered into the final model to identify factors independently associated with the client satisfaction with FP services and statistical significance was declared at a 95% CI and *p*-value < 0.05. Collinearity between variables was assessed by looking at the values of variance inflation factors (VIFs). VIF > 10 is assumed to be suggestive of the presence of multicollinearity. Hosmer-Lemeshow test was used to see model fitness at *p*-value > 0.05.

### Ethics approval and consent to participate

Ethical clearance was approved by the Haramaya University, College of Health and Medical Science, Institutional Health Research Ethics Review Committee (IHRERC). Permission letter was also obtained from each Public health facilities’ of the medical director. Moreover, informed written consent was obtained from each participant after explaining the purpose and benefits of the study. Confidentiality was kept by using a medical record number in which was immediately detached and filed separately in a confidential manner.

## Results

### Characteristics of respondents

A total of 492 FP users were successfully interviewed immediately after having received care in four (4) public health facilities and making a response rate of 98.4%.

One-hundred-six (41.8%) of respondents from the hospital were satisfied by the service provided. The mean ± SD age of respondents was 28 ± 4.8 years. Forty-four (97.8%) of the study participants who were within the age group of < 18 years of age, were dissatisfied by the service they got. From the total study participants, 107 (35.1%) of urban residents and 58 (79.5%) of rural residents were satisfied by the service provision. One hundred thirteen (81.9%) of the single study participants were dissatisfied by the service they got. From the total of study participants, about 184 (53.3%) of Muslims and 103 (70.1%) of Christians in religion were dissatisfied by the service provision. Eighty five (66.4%) and 97 (79.5%) of government employee and students were dissatisfied by the service provided. Among the respondents, 207 (69.5%) of re-visit FP clients were dissatisfied by the service provided by the health facilities. Table 1.

**Table 1:** Socio-Demographic characteristics and client satisfaction with FP services of Family planning user’s in public health facilities of Jigjiga town, eastern Ethiopia, February–March 2017 (*n* = 492)

Variables	Classification	Client satisfaction		
		Satisfied (*n* = 205)	Unsatisfied (*n* = 287)	X^2^ (*p*-value)
		N^o^ (%)	N^o^ (%)	
Age	< 18 yrs	1 (2.2)	44 (97.8)	35.5 (0.000)
	18-31 yrs	146 (48.8)	153 (51.2)	
	> 31 yrs	58 (39.2)	90 (60.8)	
Residence	Rural	58 (79.5)	15 (20.5)	50.35 (0.000)
	Urban	147 (35.1)	272 (64.9)	
Marital status	Single	25 (18.1)	113 (81.9)	108.7(.000)
	Married and live together	97 (37.9)	159 (62.1)	
	Married but not live together	44 (89.8)	5 (10.2)	
	Divorced	39 (79.6)	10 (20.4)	
Education	Don’t have regular education	58 (47.2)	65 (52.8)	66.38(.000)
	Write and read only	83 (56.1)	65 (43.9)	
	1–8	38 (39.2)	59 (60.8)	
	9–12	25 (51.0)	24 (49.0)	
	12^+^	1 (1.3)	74 (98.7)	
Religion	Muslim	161 (46.7)	184 (53.3)	25.3(.000)
	Christian	44 (29.9)	103 (70.1)	
Ethnicity	Somali	136 (42.5)	184 (57.5)	0.49 (0.78)
	Oromo	50 (39.1)	78 (60.9)	
	Others^a^	19 (43.2)	25 (56.8)	
Occupation	Government employee	43 (33.6)	85 (66.4)	72.5(.000)
	Private employee	15 (71.4)	6 (28.6)	
	Merchant	24 (100)	0 (0)	
	House wife	98 (49.7)	99 (50.3)	
	Student	25 (20.5)	97 (79.5)	

^a^1_Amhara, 2_Tigray, and 2_Gurage

All public health facilities in the study area offer three types of family planning methods; injectable Depo, Implanon, and the Oral contraceptive pill. During the data collection period, the prevalence of injectable contraceptives (Depo) was 295 (59%) and prevalence of Implanon and the oral contraceptive pill was 160 (32%) and 45 (9%) respectively. From those, 133 (86.9%) of implant users were dissatisfied by the service provision. From the total of respondents, 420 (85.4%) were agreed with the opening hour of the public health facility that they were using it. The feeling of waiting time was long among 271 (55.1%) and short for 172 (34.9%) respondents. However, only 186 (44.3%) of the study participants who were comfortable with the opening hour of the public facility were satisfied by the service.

About 97(43.9%) and 68 (30.9%) of the respondents who responded that time of the consultation with the service provider was “about right”, and “too short” were satisfied respectively. From respondents who didn’t get the method of their chose, only 4 (10%) were satisfied by the service provided. Regarding the distance to the public health facility from their home, 295(60%) of the respondents said it was taken less than 30 min, the remaining 197(40%) were responded it takes more than 30 min. From the total study participants, only 49 (25.1%) waited for 30 min- 1 h and 20 (27.4%) waited for > 1 h to get service were satisfied by the service provision. Table 2.

**Table 2:** Family planning utilization, health facility related characteristics and client satisfaction with FP services among FP user’s at public facility of Jigjiga town, eastern Ethiopia, February–March 2017 (*n* = 492)

Variables	Classification	Client satisfaction		
		Satisfied (*n* = 205) N^o^ (%)	Unsatisfied (*n* = 287) N^o^ (%)	X^2^ (*p*-value)
Frequency of Visit	Repeat	91 (30.5)	207 (69.5)	2.9 (0.31)
	New	89 (45.9)	105 (54.1)	
Type of public health facility	Karamara hospital	156 (41.8)	217 (58.2)	3.24 (0.35)
	Jigjiga health center	27 (50.0)	27 (50.0)	
	Aayardaga health center	13 (35.1)	24 (64.9)	
	Hanti Shacab health center	9 (32.1)	19 (67.9)	
Received their preferred method	Yes	201 (44.5)	251 (55.5)	17.96 (0.000)
	No	4 (10.0)	36 (90.0)	
Method that the client was using	Oral contraceptive	14 (35.0)	26 (65.0)	81.85 (0.000)
	Injectable	171 (57.2)	128 (42.8)	
	Implant	20 (13.1)	133 (86.9)	
Distance from home	< 30 min	106 (35.9)	189 (64.1)	9.9 (0.02)
	30 min-1 h	99 (50.3)	98 (49.7)	
Time of consultation	About right	97 (43.9)	124 (56.1)	39.3 (0.000)
	Too short	68 (30.9)	152 (69.1)	
	Too long	40 (78.4)	11 (21.6)	
Opening hours convenience	Yes	186 (44.3)	234 (55.7)	8.1 (0.004)
	No	19 (26.4)	53 (73.6)	
Waiting time	No wait	25 (48.1)	27 (51.9)	65.9 (0.000)
	< 30 min	111 (64.5)	61 (35.5)	
	30 min-1 h	49 (25.1)	146 (74.9)	
	> 1 h	20 (27.4)	53 (72.6)	
Feeling on waiting time	No wait	0 (0)	50 (100)	80.4 (0.000)
	Short	112 (65.1)	60 (34.9)	
	Long	93 (34.4)	177 (65.6)	

Privacy was maintained for 247 (50.4%) of the clients. From those, 148 (54.6%) of study participants were unsatisfied for service they got. Regarding the communication of the service provider, 104 (60.1%) and 68 (30.9%) of clients who responded “communication of the service provider was easily understandable” and that “difficult to understand” were satisfied. Mothers who got an advising about explain the method, side effects, explaining any danger problem, and possibility of method changing were 43(40.6%), 148(54.6%), 82(39.8%) and 117(59.45%) were satisfied by the service they got retrospectively. During the explanation of family planning methods least attention was given to spermicidal 10 (2%). Based on the study participants, methods such as diaphragm, Tubal ligation, and natural contraceptive methods were not mentioned totally. Table 3.

**Table 3:** client-provider interaction and information given by FP service provider characteristics and client satisfaction with FP services among FP user’s in public health facilities of Jigjiga town eastern Ethiopia, February–March 2017 (*n* = 492)

Variables	Classification	Client satisfaction		
		Satisfied (*n* = 205) N^o^ (%)	Unsatisfied (*n* = 287) N^o^ (%)	X^2^ (*p*-value)
Privacy was maintained	Yes	123 (45.4)	148 (54.6)	3.4 (0.064)
	No	82 (37.1)	139 (62.9)	
Information given	Explain the method	43 (40.6)	63 (59.4)	0.067 (0.795)
	Demonstrate how to use	121 (63.7)	69 (36.3)	61.7 (0.000)
	Describe side effects	148 (54.6)	123 (45.4)	41.6 (0.000)
	Explain experience any problem	82 (39.8)	124 (60.2)	0.50 (0.477)
	Explain possibility of changing	117 (59.4)	80 (40.6)	42.6 (0.000)
	Area of follow up	165 (37.8)	272 (62.2)	24.58 (0.000)
	Informed importance	205 (48.9)	214 (51.1)	2.7 (0.431)
Informed about other methods	Oral contraceptive pills	40 (16.8)	198 (83.2)	117 (0.000)
	Injectable (Depo)	103 (34.2)	198 (65.8)	4.81 (0.526)
	IUCD	24 (16.3)	123 (83.7)	
	Condom	34 (21)	128 (79)	
	Implants	49 (27.7)	128 (72.3)	
	Spermicidal	0 (0)	10 (100)	
STI Checked	No	205 (41.7)	287 (58.3)	Na
Pregnancy checked	Yes	205 (46.2)	239 (53.9)	Na
	No	0 (0)	48 (100)	
Check medical, surgical and other problem	No	205 (41.7)	287 (58.3)	Na
Asking questions	Yes	205 (42.5)	277 (57.5)	Na
	No	0 (0)	10 (100)	
Communication of the provider	Easy to understand	104 (60.1)	69 (39.9)	37.53 (0.000)
	Difficult to understand	68 (30.9)	152 (69.1)	
	Don’t understand	33 (33.3)	66 (66.7)	

Sixty two (21.1%) and 143 (72.2%) participants who had good and poor knowledge about family planning respectively were satisfied by the service they had gotten. Table 4 The findings of this study showed that the overall client satisfaction with FP services among FP users of public health facilities of Jigjiga town was 41.7% with 95% CI of (37 − 46.1%). Table 5.

**Table 4:** Interpersonal characteristics and client satisfaction with FP services among FP user’s in public health facilities of Jigjiga town, eastern Ethiopia, February–March 2017 (*n* = 492)

Variables	Classification	Client satisfaction		
		Satisfied (*n* = 205) N^o^ (%)	Unsatisfied (*n* = 287) N^o^ (%)	X^2^ (*p*-value)
Knowledge	Good	62 (21.1)	232 (78.9)	127 (0.000)
	Poor	143 (72.2)	55 (27.8)	
Get service with short period	Yes	78 (43.1)	103 (56.9)	0.2 (0.62)
	No	127 (40.8)	184 (59.2)	
Provider gives good service	Yes	181 (51.0)	174 (49.0)	45.5 (0.000)
	No	24 (17.5)	113 (82.5)	
Counseling was clear and satisfactory	Yes	98 (50.0)	98 (50.0)	9.3 (0.002)
	No	107 (36.1)	189 (63.9)	
Preference of service provider’s sex	Female	49 (22.9)	165 (77.1)	3.6 (0.27)
	Male	203 (62.1)	124 (37.9)

**Table 5:** Components of client satisfaction with FP services in public health facilities of Jigjiga town, eastern Ethiopia, February–March 2017 (*n* = 492)

Variables	Classification	Number	Percent (%)
I would like to come back to this health facility again	Agree	397	80.7
	Neutral	63	12.8
	Disagree	32	6.5
I was provided with all the information I needed	Agree	52	10.6
	Neutral	408	82.9
	Disagree	32	6.5
I would recommend this health facility to someone else	Agree	162	32.9
	Neutral	293	59.6
	Disagree	37	7.5
All my health need were met today	Agree	192	39
	Neutral	271	55.1
	Disagree	29	5.9
I felt free to ask all questions	Agree	117	23.8
	Neutral	321	65.2
	Disagree	54	11
I was treated with respect	Agree	117	23.8
	Neutral	297	60.4
	Disagree	78	15.9

### Factors associated with client satisfaction of FP services

All independent variables those were found statistically significant in chi-square (X^2^) tabulation/bivariate analysis at the *p*-value of ≤0.25 considered for multivariate regression analysis.

After the above variables (which were statistically significant in chi-square tabulation / bivariate analysis at the *P*-value of < 0.25) entered in to the multivariate regression analysis, a significant associated factors were identified at the p-value of ≤0.05. Clients who had good knowledge on FP were less likely to be satisfied with FP services compared to those who had poor knowledge [adjusted odds ratio (AOR) = 0.037, 95% CI: 0.019–0.072]. FP users who were advised on the side effect of FP that they were choosing were more satisfied with the FP service compared to those who were not advised (AOR = 2.4, 95% CI: 1.41–4.23). Table 6.

**Table 6:** Factors associated with client satisfaction of FP services among FP users in public health facilities of Jigjiga town, Eastern Ethiopia, February–March 2017 (*n* = 492)

Variable	Response	Client satisfaction		COR (95% CI)	AOR (95% CI)
		Satisfied n (%)	Unsatisfied n (%)		
Knowledge	Good	62 (21.1)	232 (78.9)	0.1 (0.07–0.16)	.037 (.019–.072)*
	Poor	143 (72.2)	55 (27.8)	1.00	1.00
Demonstrated how to use	Yes	120 (63.5)	69 (36.5)	4.6 (3.1–6.7)	16.1 (8.4–30.7)*
	No	84 (27.8)	218 (72.2)	1.00	1.00
Described side effect	Yes	148 (54.6)	123 (45.4)	3.5 (2.4–5.1)	2.4 (1.4–4.2)*
	No	57 (25.8)	164 (74.2)	1.00	1.00
Explained possibility of changing	Yes	117 (59.4)	80 (40.6)	3.4 (2.4–5)	.97 (.34–2.76)
	No	88 (29.8)	207 (70.2)	1.00	1.00
Distance home to health facility	< 30 min	106 (35.9)	189 (64.1)	0.56 (0.39–0.8)	.24 (.14–.42)*
	30 min-1 h	99 (50.3)	98 (49.7)	1.00	1.00
Provider gave good service	Yes	181 (51.0)	174 (49.0)	4.9 (3–8)	.58 (.1–3.6)
	No	24 (17.5)	113 (82.5)	1.00	1.00
Waiting time	No wait	25 (48.1)	27 (51.9)	2.5 (1.16–5.2)	.02 (.002–.18)
	< 30 min	111 (64.5)	61 (35.5)	4.8 (2.6–8.8)	.37 (.07–2.1)
	30 min- 1 h	49 (25.1)	146 (74.9)	0.9 (0.5–1.6)	.1 (.012–.92)
	> 1 h	20 (27.4)	53 (72.6)	1.00	1.00
Cleanness of the clinic	Poor	20 (8.3)	221 (91.7)	0.006 (0.001–.052)	.001 (.00–.02)
	Neutral	171 (72.5)	65 (27.5)	0.2 (0.02–1.46)	.04 (.002–.88)
	Good	14 (93.3)	1 (6.7)	1.00	1.00

*Statistically significant at *p* < 0.05 in the multivariable analysis

FP users who were shown demonstrate how to use the FP that they were choosing were more satisfied with the FP service compared to those who were not shown (AOR = 16.1, 95% CI: 8.4–30.7). The odds of distance client’s home to health facility < 30 min were lower among FP users who were satisfied with FP services compared to those who were unsatisfied (AOR = 0.24, 95% CI: 0.14–0.42). Table 6.

## Discussion

The findings of this study showed that the overall client satisfaction with FP services among FP users of public health facilities of Jigjiga town was 41.7% with 95% CI of (37 − 46.1%). Regarding the factors; knowledge on FP, demonstrate how to use the method, describing side effects of the method and distance of FP user’s home to health facility were found to be significantly associated with the client satisfaction with FP services. Table 6.

The overall client satisfaction with the family planning services was found to be 41.7%

This result is in line with a study carried out in public health facilities of West Shoa Zone, Central Ethiopia and in Villanueva de los Castillejos, Spain which was 42 and 45% [22, 23] and this is higher than a study carried out in Lahore that was 33% [24]. However, the result of this study is also lower than those of studies conducted among FP users in central Ethiopia, BahrDar, Bangladesh, Hosaena, Kenya, Nigeria, Mozambique, Tanzania and Port Said city which were, 62.6, 66.1%, 75, 75.3%, 81, 85, 86, 91 and 95.4% respectively [13, 14, 18–20, 25–27]. This lower level of client satisfaction may be related to the low quality of FP service provision on those public health facilities. The other reason may be this study didn’t consider the private and non-governmental health facilities compared to the above some studies. Regarding different studies, the level of client satisfaction is expected to be higher in private health facilities [28].

A thorough review of previous studies has shown that client satisfaction among FP users is complex and bounded by many factors. Among the information given factors; demonstrate how to use the method and describe the side effect of the method were found to be significantly associated with client satisfaction. In this study, FP users who were advised on the side effect of FP and FP users who were shown demonstrate how to use the FP that they were choosing were more satisfied with the FP service compared to those who were not. This result is similar to the studies carried out in Ethiopia and the USA [22, 29]. This finding might be related to the fact that the information imparted during service contact that enables clients to choose and employ contraception with satisfaction [15]. Clients lack information results in a negative attitude towards methods whenever they experience the problems. This might increase the degree of dissatisfaction and finally, the client might discontinue the FP method.

Regarding interpersonal characteristics, participants who had good knowledge of FP were less likely to be satisfied with FP services compared to those who had poor knowledge. 232 (78.9%) of who had a good knowledge were dissatisfied; and 143 (72.2%) of mothers who had poor knowledge were satisfied by the service they got. This is different from other studies [30, 31]. A reason for this is might be, having good knowledge of the service may dissatisfied clients if the quality of service is poor.

The odds of distance client’s home to health facility < 30 min were lower among FP users who were satisfied with FP services compared to those who were unsatisfied. This is different from the study performed in Malawi [32]. This needs further study but, it might be clients who get the service by going long distances may satisfy easily compared to those who get the service nearly.

### Strengths and limitations of the study

Client satisfaction is a basic element to clients’ decisions to use and to continue the service for their future time. And it is a core indicator of the quality of service. However, assessing satisfaction could be a complex concept. This study tried to examine different potential factors for the service. However, since this study conducted a public health facility based, dissatisfied women might be at their home. Respondents usually would not want to express negative feelings to unknown persons on their level of satisfaction, and this could be overestimated of the level of satisfaction with FP services.

## Conclusions

The overall client satisfaction with FP services was relatively low, which might impact on the quality of the service and utilization of FP methods. The variables found to be significantly associated with the client satisfaction were knowledge of FP, demonstrate how to use the method, describing the side effect of the method and distance of FP user’s home to the health facility. It needs improvements in client interaction especially on the information given, and it needs a qualitative study on the association of knowledge and client satisfaction.

## Data Availability

All the data supporting the findings is contained within the manuscript, when there is in need the data-set used for the present study’s conclusion can be accessible from the corresponding author on reasonable request.

## Abbreviations

AOR: Adjusted Odds Ratio
CI: Confidence interval
COR: Crude Odds Ratio
CSA: Central Statistical Agency
FP: Family Planning
IHRERC: Institutional Health Research Ethics Review Committee
SPSS: Statistical Package for Social Science
VIF: Variance inflation factors
